# Multidimensional Profiling of Senescence in Eastern Honey Bee, *Apis cerana* (Hymenoptera: Apidae), Workers: Morphology, Microstructure, and Transcriptomics

**DOI:** 10.3390/insects16090902

**Published:** 2025-08-28

**Authors:** Qiang Ma, Zachary Y. Huang, Qianmin Hai, Jun Zhang, Xiangyou Tang, Xiaoqun Dang, Jinshan Xu, Zhengang Ma, Zeyang Zhou

**Affiliations:** 1 Key Laboratory of Pollinator Resources Conservation and Utilization of the Upper Yangtze River, Ministry of Agriculture and Rural Affairs, Chongqing Normal University, Chongqing 401331, China; maqiang@cqtgmc.edu.cn (Q.M.); 2024110513017@stu.cqnu.edu.cn (Q.H.); 2023110513036@stu.cqnu.edu.cn (J.Z.); 2023010513010@stu.cqnu.edu.cn (X.T.); xqdang@cqnu.edu.cn (X.D.); xujinshan2003@aliyun.com (J.X.); 2 Department of Basic Medicine, Chongqing Three Gorges Medical College, Chongqing 404120, China; 3 Chongqing Key Laboratory of Vector Control and Utilization, College of Life Sciences, Chongqing Normal University, Chongqing 401331, China; 4 Department of Entomology, Michigan State University, East Lansing, MI 48824, USA; bees@msu.edu

**Keywords:** eastern honey bee, aging, morphological character, structural features, transcriptomics

## Abstract

Worker honey bees are crucial for colony stability and ecosystem pollination. However, the cross-scale aging features and underlying mechanisms in the Eastern honey bee (*Apis cerana*) remain poorly understood. This study systematically investigated age-related changes in *A. cerana* workers across youth (1~5 days post-emergence, dpe), middle age (29 dpe), and old age (50 dpe) through integrated morphological, ultrastructural, and transcriptomic analyses. With increasing age, the phenotypic deterioration in the old bees (OBs) was significant: the body color brightness decreased by 16.7% compared to the young bees (YBs) (*p* < 0.001), and the hair density of the head, thorax, and abdomen declined by 63.5%, 97.2%, and 91.5%, respectively (*p* < 0.0001). The wing wear index (WWI) increased to 96.7% (*p* < 0.0001). The locomotor performance declined sharply, with only 6.7% of the OBs successfully reaching the feeding platform within 15 s (*p* < 0.0001). Ultrastructural analysis revealed sensory organ abrasion, flattened thoracic bristles, thickened cuticle, and 90.4% increased mitochondrial damage (*p* < 0.0001). The autophagosomes showed dynamic changes, with 81.8% reduction versus those of mid-aged bees (MBs) (*p* < 0.001), which suggests that mitochondrial dysfunction and autophagy dysregulation may be the core driving factors behind aging. Transcriptomics identified 67 differentially expressed genes enriched in lifespan regulation, glutathione metabolism, and lysosomal pathways. Fifteen key aging-related genes were identified, such as *major royal jelly protein 3* (*MRJP3*), *synaptic vesicle glycoprotein 2A* (*SV2A*), and *apidermin 3* (*APD3*), whose expression dynamics have been shown to be closely related to nutritional metabolism, behavioral perception, and the decline of epidermal barrier function. This work establishes the first multidimensional aging evaluation system for *A. cerana*, providing critical insights into bee senescence mechanisms and colony health optimization.

## Simple Summary

Worker bees are essential for maintaining healthy honey bee colonies, which play a vital role in pollinating crops and sustaining ecosystems. However, premature or dysregulated senescence of these bees disrupts colony balance and reduces pollination efficiency, which may contribute to colony decline under environmental stress and thus pose risks to agriculture and biodiversity. This study investigated how aging affects key pollinators by examining changes in their bodies, internal structures, and genes across three life stages: youth, middle age, and old age. Older bees showed visible signs of aging, such as a faded body color, loss of body hairs, and worn wings, along with reduced movement and damage to cellular components like mitochondria. Genetic analysis revealed key genes linked to energy metabolism, antioxidant defenses, and skin protection that decline with age. These findings help explain why older bees struggle to perform tasks and highlight potential targets for interventions to slow aging. By creating a comprehensive evaluation system, this research offers practical insights for improving hive management strategies, safeguarding pollinator populations, and ensuring stable crop pollination services—critical steps for protecting ecosystems and supporting food security.

## 1. Introduction

Honey bees (genus *Apis*), as the most efficient pollinators in terrestrial ecosystems, play an irreplaceable role in maintaining biodiversity, ensuring agricultural productivity, and driving global economic development by facilitating cross-pollination for over 75% of major food crops and 90% of wild plants [1,2]. Statistics indicate that honey bee pollination contributes USD 235~577 billion annually to global food production, representing > 80% of the total economic value generated by insect pollinators [3,4], and their derivative products (such as honey and propolis) have widespread applications in the food industry and biomedical field, which further highlights their multidimensional industrial value [5]. Most of the aforementioned statistics were for the western honey bee, *Apis mellifera* due to its worldwide distribution. Within the Asian ecological fauna, *Apis cerana* stands out as a key species maintaining regional ecological balance, thanks to its high adaptability to native plants, resistance to mites, and tolerance to low temperatures [6,7,8]. However, in recent years, habitat loss, pesticide exposure, and cross-species transmission of pathogens have led to a sharp decline in the population of both species [9,10]. Among these threats, premature aging of worker bees causing colony losses has emerged as a critical factor threatening the sustainability of bee colonies [11,12,13]. While aging has been studied in *A. mellifera* [14,15], few studies have used *A. cerana*. Thus, the mechanisms driving the senescence of *A. cerana* worker bees, from behavioral decline to molecular regulation, remain unclear, which hinders the development of targeted intervention strategies for colony losses.

As typical eusocial insects, the lifespan of worker bees of *A. mellifera* is closely related to their labor division, with worker bees of different ages undertaking distinct tasks and exhibiting a unique pattern of “task-dependent senescence”. For instance, younger worker bees are responsible for brood care within the hive, while middle-aged bees shift to high-risk foraging activities outside [16]. This labor strategy and life-history trade-off make them an ideal model for studying social aging [17]. Previous research has primarily focused on the molecular mechanisms of aging in *A. mellifera*, discovering that juvenile hormone (JH) and vitellogenin (Vg) play crucial roles in regulating their lifespan [18,19,20]. The JH levels are positively correlated with the transition to foraging behavior and aging in worker bees, where higher JH titers accelerate the progression to older roles [14], while Vg not only participates in reproductive regulation but is also closely associated with lifespan extension in *A. mellifera* [20]. However, *A. cerana* may exhibit unique adaptations in its immune response and energy metabolism under specific environmental pressures [21,22,23], despite sharing conserved features in its labor division scheduling and JH regulation with *A. mellifera* [24,25]. These observations suggest potential divergences in aging-related regulatory networks that warrant further investigation. Notably, although studies have examined the physiological and biochemical changes associated with aging in bees, most have concentrated on a single aspect, such as alterations in certain enzyme activities or fluctuations in antioxidant levels [26,27], lacking a systematic, multidimensional integrated analysis that can reveal the causal relationship between organ functional decline and gene expression regulation. We hypothesized that senescence in *A. cerana* workers manifested as progressive multiscale degradation, driven by mitochondrial dysfunction, autophagy dysregulation, and downregulation of key genes involved in metabolism and stress response.

In light of this, the present study aims to fill this gap by systematically and comprehensively identifying the aging characteristics of *A. cerana* worker bees through the integrated use of stereomicroscopy, scanning electron microscopy (SEM), transmission electron microscopy (TEM), and transcriptome sequencing techniques. This study aims to establish a multidimensional evaluation system for social insect aging, providing a theoretical basis for optimizing hive health management strategies and advancing research in the biology of aging.

## 2. Materials and Methods

### 2.1. Rearing and Sample Collection of Apis cerana

The *A. cerana* used in this study originated from healthy bee colonies maintained at the Beekeeping Base of Chongqing Normal University (29.36° N, 106.17° E) during the spring of 2024. All experimental procedures adhered to internationally accepted ethical guidelines for invertebrate experiments [28,29]. To collect worker bees of different ages, brood combs with consistent capping dates (20th day of worker bee development) were selected and transferred to a temperature- and humidity-controlled incubator (TAISITE HWS-250P, Tianjin, China), at a temperature of 35.0 ± 0.5 °C and relative humidity of 55 ± 5%, and maintained in darkness to induce emergence. Newly emerged worker bees with an eclosion time of <24 h were collected the following day. Newly emerged bees were then marked with a non-toxic oil-based marker (POSCA 0.7 mm, Shinagawa, Japan). A total of 1200 bees (400 per colony × 3 colonies) were marked to ensure ≥60 viable individuals per age group at final sampling (see Table S1 for the survival rates). Marked bees were returned to source hives. They were later captured at 29 days and 50 days post-eclosion (dpe). Based on their post-eclosion age, worker bees were grouped and named as young bees (YBs, 1~5 dpe), mid-aged bees (MBs, 29 dpe, representing the foraging phase), and old bees (OBs, 50 dpe, near the end of their lifespan) (see Figure S1 for the experimental design). For transcriptome sequencing samples and qRT-PCR validation, three independent biological replicates (each from a different colony) were established for each age group in two parallel cohorts, totaling 18 samples (with n = 3 bees’ heads per sample). All samples from both cohorts were rapidly frozen in liquid nitrogen and stored at −80 °C.

### 2.2. Observation of Morphological Characteristics

Twenty healthy and physically intact worker bees were randomly selected from each age group as observation samples. A stereo microscope (Leica S9E, Wetzlar, Germany) was used to observe and document morphological indicators such as body color, hair density, and wing integrity across the different age groups.

#### Measurement of Dorsal Brightness

Dorsal brightness was measured on the thoracic tergum of each worker bee. Images of the dorsal region were captured using the built-in imaging system of the Leica S9E stereo microscope (coupled with Leica Application Suite X software, version 5.3.1). Light conditions were standardized during image acquisition: light intensity was set to 5000 lux, exposure time was set to 1/100 s, and the distance between the sample and the lens was fixed at 10 cm to avoid luminance variations. Luminance was quantified using Image J 1.50 software (NIH, USA) by extracting the mean gray value (ranging from 0 to 255) of the dorsal region in each image, with higher gray values indicating greater brightness.

The observation results were quantitatively analyzed using Image J 1.50 software, including the calculation of optical density values and the counting number of hairs per square millimeter (mm^2^). Additionally, the wing wear index (WWI) was calculated to quantify the integrity of the bees’ wings. The calculation of the WWI followed the method described by Vanengelsdorp et al. [30], with wear levels classified as 0 (no wear), 1 (minor), 2 (moderate), and 3 (severe). The formula used was as follows: WWI = Σ (Wear Level × Number of Individuals)/Total Sample Size.

### 2.3. Assessment of Locomotive Abilities

A custom-made device for assessing motor abilities was constructed, referencing the methods of Peleg et al. [31]. Two disposable transparent beverage cups with a 95 mm diameter were selected, and uniform circular holes were cut into the sidewalls of each cup to ensure adequate ventilation. Subsequently, two sponge balls soaked in a 50% sugar solution were fixed to the bottom of one cup, serving as a feeding platform for the worker bees. This cup was then inverted onto the other cup, with a layer of 0.1 mm thick transparent plastic film placed in between to prevent the bees from entering the feeding area prematurely. During the experiment, 20 worker bees (with three biological replicates, totaling 60 bees) from different age groups were subjected to a hunger treatment for 2 h in a standard rearing environment with a temperature of 35 °C and a relative humidity of 55%, in constant darkness. Following the hunger treatment, the bees were instantaneously anesthetized with CO_2_ and transferred to the lower cup. Once the bees in each group had recovered, the plastic film covering the cup opening was quickly removed, and a timer was set for 15 s. During this period, the bees, driven by hunger, would crawl or fly to the feeding platform on the bottom of the upper cup to feed. At the 15 s endpoint, a rigid cardboard divider was swiftly inserted between the cups to immobilize the bee’s position. The number of bees on the feeding platform within this time frame was recorded and compared to the total number of bees, serving as an assessment of their motor abilities.

### 2.4. Scanning Electron Microscopy (SEM) Observation

Three healthy and morphologically intact worker bees were randomly selected from each age group as observation samples. On a sterile workstation, fine tweezers and dissecting scissors were used to carefully isolate the entire head, thorax, and abdomen (with visceral tissues removed) of each individual worker bee. The dissected tissue samples were immersed in fixative containing 2.5% glutaraldehyde and fixed at 4 °C for 12 h to stabilize the microscopic structures of the samples. After fixation, the samples were dehydrated using ethanol solutions of different concentrations (30%, 50%, 70%, 80%, 90%, 95%, and 100%) sequentially, with each concentration applied for 15 min to ensure complete replacement of water in the samples. The dehydrated samples were transferred to a critical point dryer (AutoSamdri 815B, Tousimis, Rockville, MD, USA) for drying. The dried samples were adhered to stubs using conductive glue and subsequently coated with gold using an ion sputter coater (Hitachi MSP-1S, Chiyoda City, Japan). The microscopic structural features of the samples were observed under a scanning electron microscope (SEM, Hitachi S-3400N-II, Chiyoda City, Japan) at an accelerating voltage of 5.00 kV.

### 2.5. Transmission Electron Microscopy (TEM) Observation

Three healthy and morphologically intact worker bees were randomly selected from each age group and anesthetized with CO_2_. The bees were first placed ventrally on wax plates and fixed onto the plates by inserting two insect pins obliquely through their thoraxes. Under a stereomicroscope, the chitinous exoskeleton of the head was scored open with fine tweezers to completely dissect out the brain, which was then immediately transferred to pre-cooled 2.5% glutaraldehyde fixative and fixed at 4 °C for 24 h. Subsequently, the brain tissues were refixed in 1% osmium tetroxide solution for 2 h. After fixation, the samples were rinsed three times with 0.1 mol/L phosphate-buffered saline (PBS, pH 7.4). They were then dehydrated in acetone solutions of increasing concentrations (30%, 50%, 70%, 80%, 90%, 95%, 100%, 100%, and 100% again), with each concentration applied for 15 min. The samples were sequentially infiltrated with a mixture of dehydrant and Epon-812 embedding medium at decreasing dehydrant ratios: 3:1 (dehydrant:Epon) for 2 h, 1:1 for 4 h, and 1:3 overnight. The samples were then transferred to pure Epon-812 embedding medium and polymerized at 60 °C in an oven for 48 h to complete the embedding process. Using an ultramicrotome (Leica UC7rt, Wetzlar, Germany), the embedded samples were sectioned into ultrathin slices with a thickness of approximately 60~90 nm, which were then mounted on copper grids. At room temperature, the sections were first stained with 2% uranyl acetate for 10~15 min and then with lead citrate for 1~2 min. The stained sections were placed under a transmission electron microscope (TEM, JEOL JEM-1400FLASH, Akishima, Japan) to observe ultrastructural features such as mitochondrial damage and the number of autophagosomes.

### 2.6. Transcriptome Sequencing and Analysis

For transcriptome sequencing, three independent biological replicates (each from a different colony) were set up for each age group, totaling nine samples. All samples were rapidly frozen in liquid nitrogen and stored at −80 °C. Total RNA was extracted from each sample (with 3 bees’ heads) using Trizol Reagent (Invitrogen, Waltham, MA, USA) according to the manufacturer’s instructions. Differential gene expression libraries were constructed and analyzed by Novogene Corporation (Beijing, China). The raw sequencing data for the head tissues of worker bees from different age groups of the *A. cerana* can be found at PRJNA1234368.

Based on the transcriptome sequencing data, NovoMagic cloud platform (V2.0, https://magic-plus.novogene.com) was used to screen for differentially expressed genes (DEGs). Differential analysis between groups was conducted using the DESeq2 algorithm (v1.38.3), with a fold-change ≥ 2 and a false discovery rate (FDR) threshold ≤ 0.05. Functional annotation was performed for significant DEGs, followed by Gene Ontology (GO) functional enrichment and Kyoto Encyclopedia of Genes and Genomes (KEGG) pathway enrichment analysis using tools on the NovoMagic website. Hierarchical clustering analysis of the FPKM values of the genes was conducted using mainstream clustering tools on the NovoMagic website, with rows normalized using the Z-score method. Genes with similar expression patterns were clustered to deeply explore gene functions and identify key molecular targets related to aging, laying the foundation for subsequent in-depth studies on the aging mechanism of *A. cerana*.

### 2.7. Validation of DEGs by qRT-PCR

For qRT-PCR validation, sample collection and RNA extraction followed the same protocol as described for transcriptome sequencing (Section 2.6), with three biological replicates (from 3 different colonies) and 3 bees’ heads per replicate per age group. Total RNA was reverse-transcribed into cDNA using a SuperScript™ IV kit (Thermo Fisher Scientific, Waltham, MA, USA), and the obtained cDNA was stored at −20 °C for future use. Primers were designed to target 15 aging-related genes, which were screened from transcriptomic data, using β-actin as the reference gene (Table S2 for primer sequences). Following the manufacturer’s instructions, the quantitative real-time PCR (qRT-PCR) reactions were prepared in a 20 μL volume, containing 10 μL of 2× SYBR Green Master Mix (Novogene, Beijing, China), 1 μL of cDNA, and 0.4 μL each of the gene-specific primers. These reactions were run on a CFX96 Touch system (Bio-Rad, Hercules, CA, USA) under the following conditions: 95 °C for 30 s, 95 °C for 10 s, and 60 °C for 10 s, for 39 cycles. Melting curve analyses were done following the amplification cycles in order to examine the specificity of the reactions. Three technical replicates per sample were analyzed, and the relative expression levels of the genes were calculated using the 2^−ΔΔCt^ method [32].

### 2.8. Statistical Analysis

The results were analyzed using GraphPad Prism 6.02 software (GraphPad Inc., San Diego, CA, USA). Data were presented as the mean ± standard error of the mean (SE). Prior to statistical analysis, all continuous variables were tested for normality using the Shapiro–Wilk test and for homoscedasticity using Levene’s test. For variables that met the assumptions of normal distribution (Shapiro–Wilk, *p* > 0.05) and homoscedasticity (Levene’s test, *p* > 0.1), statistical differences among groups were evaluated by one-way ANOVA, followed by Tukey’s honestly significant difference (HSD) post hoc test for multiple pairwise comparisons when ANOVA indicated a significant effect. For percentage data, arcsine square-root transformation was applied prior to analysis to meet parametric test assumptions. When accounting for colony effects, a linear mixed model (LMM) with colony as a random effect was used, followed by Tukey’s HSD test for pairwise comparisons. For discrete variables that did not meet the assumptions of parametric tests, generalized linear models (GLMs) with appropriate link functions were used. Statistical significance was defined as *p* < 0.05, denoted in figures by asterisks: * *p* < 0.05, ** *p* < 0.01, *** *p* < 0.001, **** *p* < 0.0001.

## 3. Results

### 3.1. Age-Dependent Morphological Degeneration in Worker Bees

**Figure 1:**
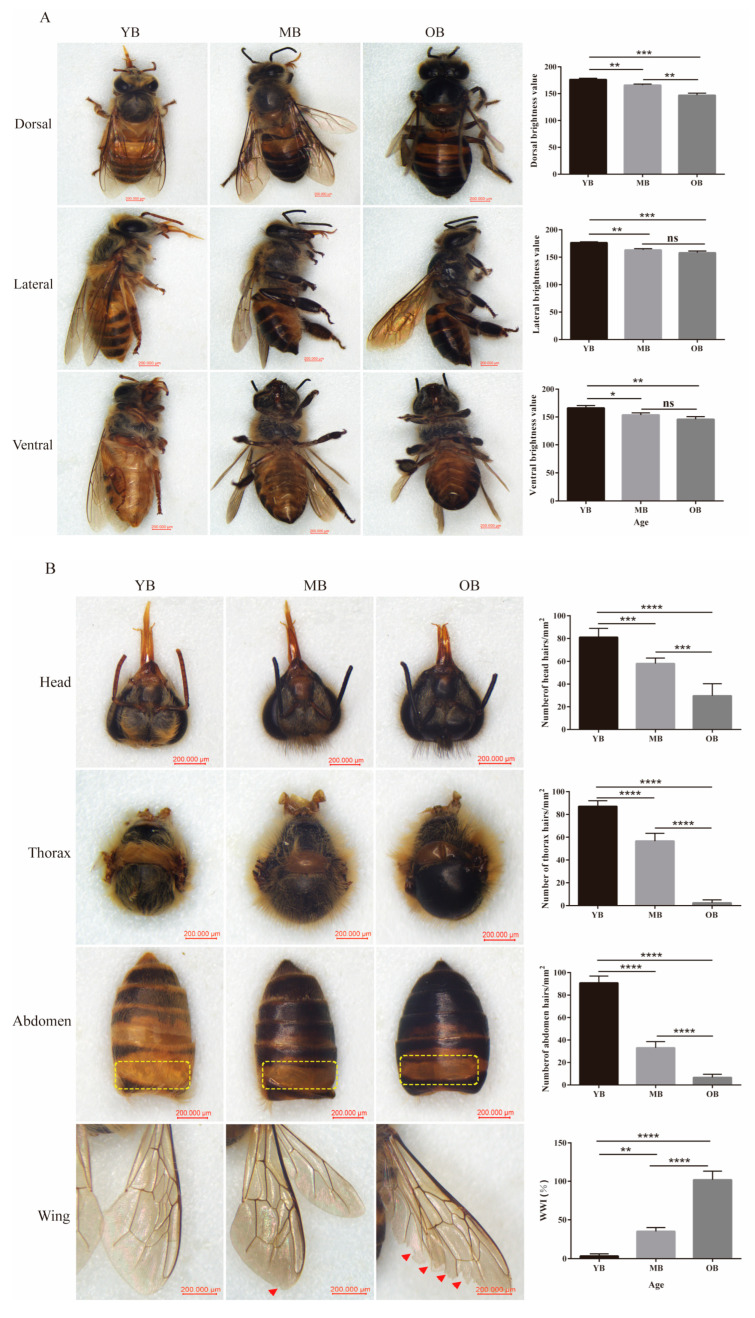
Comparison of morphological characteristics of worker bees at different ages. (**A**) Body color and brightness for differently aged worker bees from the dorsal, lateral, and ventral views. (**B**) Hair density on the head, thorax, and abdomen and WWI of worker bees at different ages. Red arrows indicate wing wear, which is more pronounced in the OBs. The yellow dashed boxes indicate the distribution of subcutaneous fat granules in the first abdominal segment. Data were presented as mean ± SE (n = 20). * *p* < 0.05, ** *p* < 0.01, *** *p* < 0.001, **** *p* < 0.0001. ns indicates not significant.

As shown in Figure 1, the morphological characteristics of *A. cerana* workers exhibited significant progressive decline with increasing age.

The YBs displayed bright body coloration (dorsal brightness: 176.3 ± 1.46 SE) (Figure 1A), dense cuticular hairs (head: 81.0 ± 3.52 hairs/mm^2^; thorax: 87.0 ± 2.24 hairs/mm^2^; abdomen: 89.8 ± 2.75 hairs/mm^2^), and intact wings (WWI: 3.33% ± 1.67) (Figure 1B). Furthermore, abundant fat body deposition was observed beneath the cuticular layer of the first abdominal segment in the YBs (Figure 1B, yellow dashed box). The MBs showed a significant reduction (6.0%) in dorsal brightness compared to the YBs (165.7 ± 1.24, *p* < 0.01) (Figure 1A), accompanied by significant decreases in hair density: 28.4% on the head (58.0 ± 2.21 hairs/mm^2^, *p* < 0.001), 34.9% on the thorax (56.6 ± 3.06 hairs/mm^2^, *p* < 0.0001), and 63.3% on the abdomen (33.0 ± 2.53 hairs/mm^2^, *p* < 0.0001). They also exhibited significantly increased wing damage (WWI: 35.0% ± 2.89, *p* < 0.01) (Figure 1B).

The OBs exhibited severe senescence, with their body color luminance being significantly decreased by 16.7% (146.9 ± 2.32, *p* < 0.001) (Figure 1A), the hair densities on their head, thorax, and abdomen dropping sharply by 63.5%, 97.2%, and 91.5% respectively (all *p* < 0.0001), and the WWI reaching 96.7% ± 4.71 (*p* < 0.0001) (Figure 1B). At the same time, the subcutaneous fat bodies in the OBs gradually decreased with age and even completely disappeared.

### 3.2. Age-Dependent Decline in Locomotor Performance of Worker Bees

**Figure 2:**
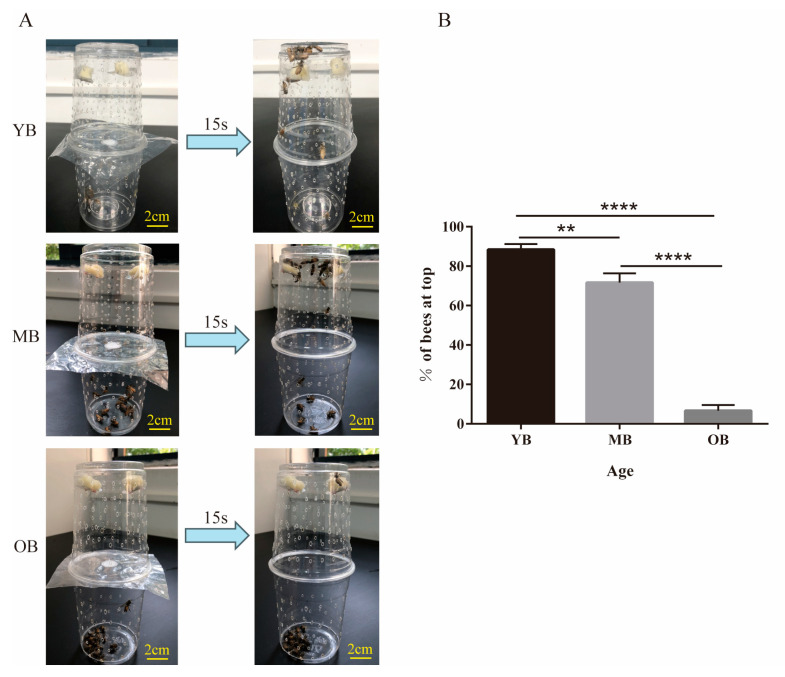
Assessment of mobility in worker bees of different age groups. (**A**) Experimental setup to measure honey bee worker mobility. (**B**) Percentage of bees reaching the top cup after 15 s in the three differently aged groups of bees. Data from three independent colonies were pooled and presented as mean ± SE (total n = 60; 20 bees per colony). ** *p* < 0.01, **** *p* < 0.0001.

There were significant differences in the number of worker bees from different age groups that reached the upper feeding platform within 15 s after being subjected to the same hunger conditions (Figure 2A). The YBs and MBs exhibited stronger mobility, with a significantly higher proportion of individuals rapidly crawling or flying to the feeding platform within 15 s compared to the OBs (*p* < 0.01). Specifically, 88.3% of the YBs successfully reached the feeding platform within 15 s, while 71.7% of the MBs did so. In contrast, the OBs exhibited decreased mobility, with only 6.7% of individuals reaching the feeding platform (Figure 2B).

### 3.3. Age-Dependent Degradation of Cuticular Structures in Worker Bees

**Figure 3:**
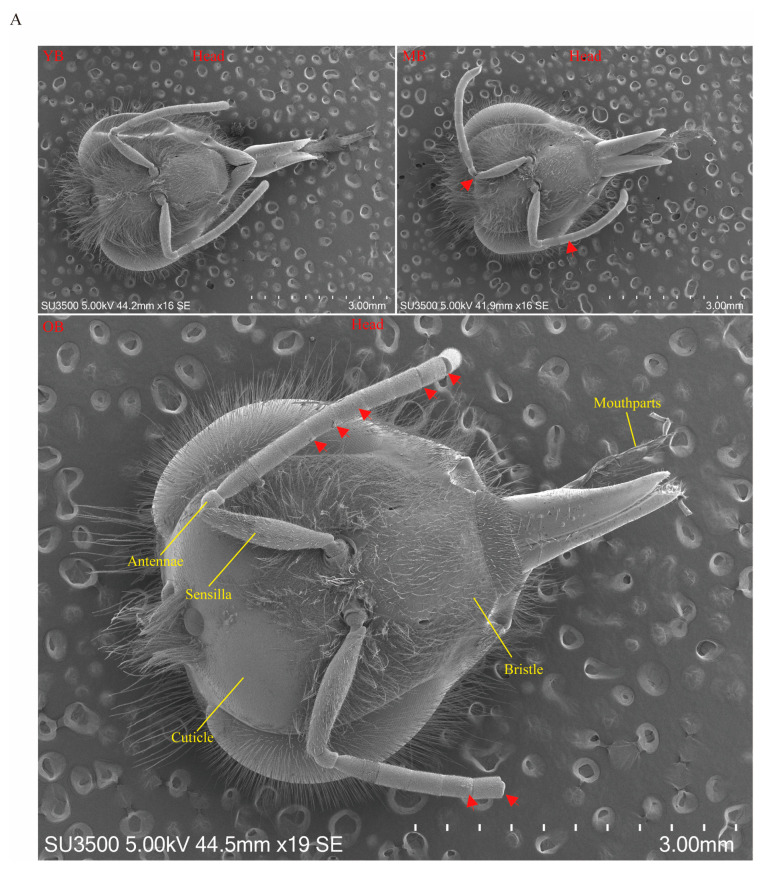

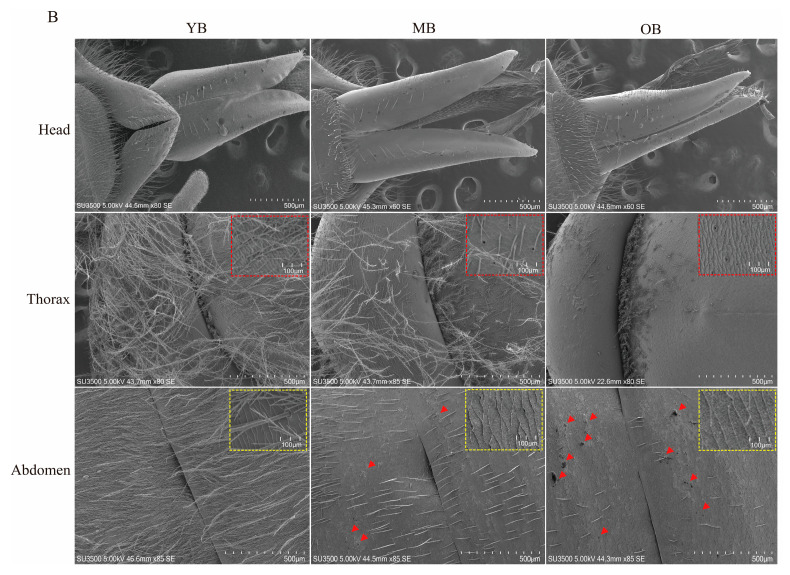
SEM observation of structural characteristics of worker bees at different ages. (**A**) Overall view of the head structures of worker bees at different ages. The yellow solid lines indicate the relevant structures of the head, including antennae, sensilla, cuticle, bristle, and mouthparts, with red arrows indicating antennae damage. (**B**) Magnified view of local structures of the head, thorax, and abdomen of differently aged worker bees. The red dashed boxes indicate epidermal pores, the yellow dashed boxes indicate the degree of epidermal roughness, and the red arrows indicate skin damage.

The body surface structure of the worker bees underwent significant morphological changes with age as shown by SEM observations (Figure 3).

Head characteristics: the head surface of the YB was structurally intact, with no apparent damage to sensory organs and mouthparts; slight wear begins to appear on the heads of the MB; the head structures of the OBs exhibited severe wear, with partial damage to their sensory organs and mouthparts (Figure 3A, red arrow).

Thoracic changes: the mesoscutum of the YBs was covered with dense villi; the villus density decreased in the thoracic region of the MBs, with holes that were approximately 2~5 μm in diameter visible on the mesoscutum surface (dashed red box in Figure 3B), whereas the OBs showed a significant loss of thoracic villi, particularly on the mesoscutum, dorsal sulcus, and metanotum, with numerous holes of the same size as those in the MBs left on the exposed cuticle surface, which suggests that long-term mechanical friction might lead to epidermal structural degeneration (Figure 3B).

Abdominal epidermal characteristics: the setae of the YBs were neatly arranged with a high villus density, clear epidermal texture, and smooth surface; the setae of the MBs showed lodging, sparse villi, and increased epidermal roughness accompanied by punctate damage (dashed yellow box and red arrow in Figure 3B); and the OBs exhibited a substantial loss or complete lodging of their setae, with numerous folds and large areas of damage on the skin surface (red arrow in Figure 3B).

### 3.4. Age-Dependent Ultrastructural Alterations in the Brain Tissue of Worker Bees

**Figure 4:**
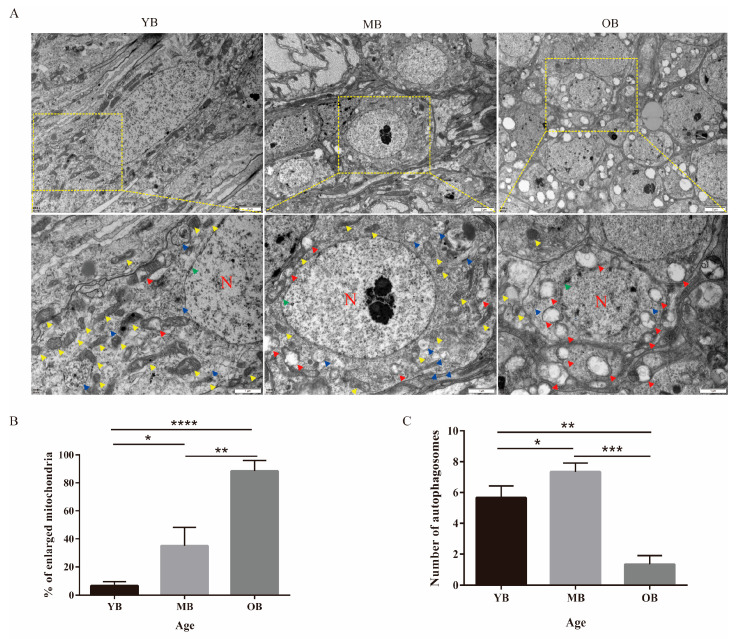
TEM observation of ultrastructural features in head tissues of worker bees in different age groups. (**A**) TEM observation of ultrastructural characteristics of mitochondria and autophagosomes in head tissues of worker bees at different stages. Yellow arrows indicate normal mitochondria, red arrows indicate damaged mitochondria, blue arrows indicate autophagosomes, green arrow indicates nuclear membrane, and N denotes the nucleus. (**B**) Mitochondrial damage in head tissues of worker bees at different ages. Five random fields of view were selected to calculate the percentage of damaged mitochondria per field of view. (**C**) Number of autophagosomes in head tissues of worker bees at different ages. Five random fields of view were selected to count the number of autophagosomes. Data are presented as mean ± SE (n = 5). * *p* < 0.05, ** *p* < 0.01, *** *p* < 0.001, **** *p* < 0.0001.

To initially evaluate whether cerebral atrophy occurs during the aging process, we measured the brain sizes of worker bees at different life stages using a stereomicroscope. The results indicated that there were no significant differences in overall brain size among the YBs, MBs, and OBs (Figure S2, *p* > 0.05). However, considering that functional senescence may precede macroscopic morphological changes [14], we further utilized TEM to observe the ultrastructural characteristics of the brain tissue in worker bees, and statistical analysis was performed on mitochondrial damage and the number of autophagosomes (Figure 4). The results showed that the mitochondria of the YBs had intact structures with the lowest damage rate (Figure 4B, damage rate: 8.3 ± 1.7%). Occasionally, fuzzy cristae structures were observed (Figure 4A). The MBs exhibited moderate mitochondrial damage (Figure 4B, damage rate: 35.0 ± 7.6%), characterized by a decreased matrix electron density and localized vacuolization (Figure 4A), and accompanied by a 1.2-fold increase in autophagosome count versus the YBs (Figure 4C, *p* < 0.05). In contrast, the OBs demonstrated severe mitochondrial degenerative changes (damage rate: 86.7 ± 3.3%), including widespread mitochondrial swelling, cristae rupture, and membrane structure disintegration (Figure 4A). The count of autophagosomes in this group was significantly decreased compared to both the YBs and MBs (Figure 4C, *p* < 0.01, *p* < 0.001). Furthermore, regarding nuclear morphology and nuclear membrane integrity, the YBs and MBs showed distinct nuclear membrane boundaries and normal nuclear morphology, while the OBs exhibited blurred nuclear membrane edges accompanied by obvious nuclear shrinkage and chromatin margination (Figure 4A, green arrow).

### 3.5. Transcriptomic Analysis of Aging-Related Genes

#### 3.5.1. Screening of Differentially Expressed Genes (DEGs) Related to Aging

**Figure 5:**
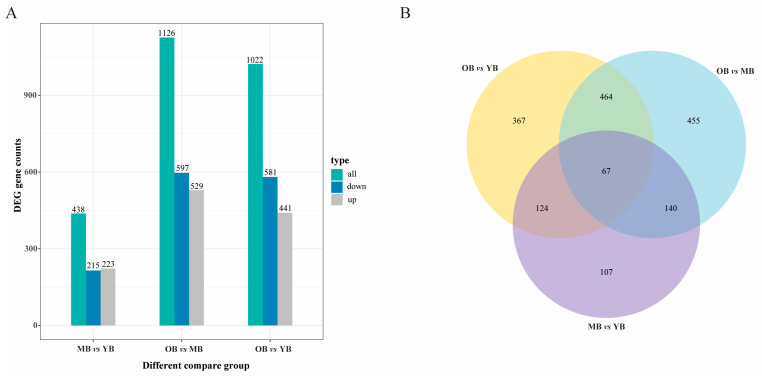
Analysis of DEGs in worker bees of different ages. (**A**) Number of DEGs in different comparisons: YBs vs. MBs, MBs vs. OBs, and YBs vs. OBs. Blue and gray represent upregulated and downregulated DEGs, respectively, with the numbers on the bars indicating the count of DEGs. (**B**) Venn diagram of DEGs for the three comparisons. Different colors represent different comparison combinations.

A total of 2586 DEGs were identified among the 10,175 transcripts that were covered. Specifically, when comparing the MBs with the YBs, 438 DEGs were found (including 223 upregulated genes and 215 downregulated genes); when comparing the OBs with the MBs, 1126 DEGs were identified (including 529 upregulated genes and 597 downregulated genes); and when comparing the OBs with the YBs, 1022 DEGs were observed (including 441 upregulated genes and 581 downregulated genes) (Figure 5A). Venn diagram analysis identified 67 overlapping DEGs across all three pairwise comparisons (MBs vs. YBs, OBs vs. MBs, OBs vs. YBs) (Figure 5B).

#### 3.5.2. Functional Enrichment Analysis of Aging-Related DEGs

**Figure 6:**
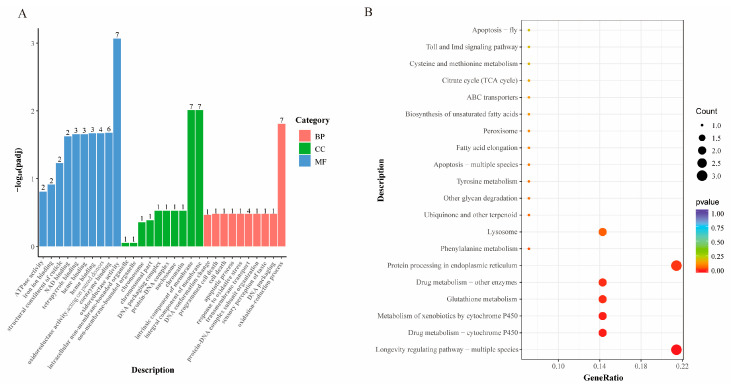
GO and KEGG enrichment analysis of common DEGs in comparison combinations of worker bees of different age groups. (**A**) Results of GO functional enrichment analysis, presented by categories of biological process (BP), cellular component (CC), and molecular function (MF). The *x*-axis represents functional descriptions, and the *y*-axis, −log_10_(*p*-value), reflects the significance of enrichment, with larger values indicating greater significance. (**B**) Results of KEGG enrichment analysis. The *x*-axis, GeneRatio, represents the ratio of the number of DEGs enriched in a particular pathway to the total number of DEGs. The *y*-axis lists the pathway names. The color of each dot indicates the *p*-value (redder colors indicate greater significance), and larger size of each dot represents a higher number of genes enriched in that pathway.

GO and KEGG enrichment analyses were conducted on the common DEGs from the three comparison combinations: MBs vs. YBs, OBs vs. MBs, and OBs vs. YBs (Figure 6). The results of the GO functional enrichment showed that these common DEGs are mainly involved in biological processes such as redox reactions and transmembrane transport, cellular components such as biological membranes, and molecular functions such as oxidoreductase activity (Figure 6A). The KEGG enrichment analysis revealed that these genes are primarily enriched in signaling pathways such as longevity regulation, drug metabolism, xenobiotic metabolism, tyrosine metabolism, glutathione metabolism, lysosomal, and endoplasmic reticulum protein processing (Figure 6B). These pathways are closely related to worker bees’ lifespan, metabolic conversion of xenobiotics, melanin synthesis, oxidative stress damage, processing of metabolic waste, and protein processing and modification. These findings suggest that these genes may play crucial regulatory roles in the aging process of worker bees.

#### 3.5.3. Identification of Genes Related to Aging

**Figure 7:**
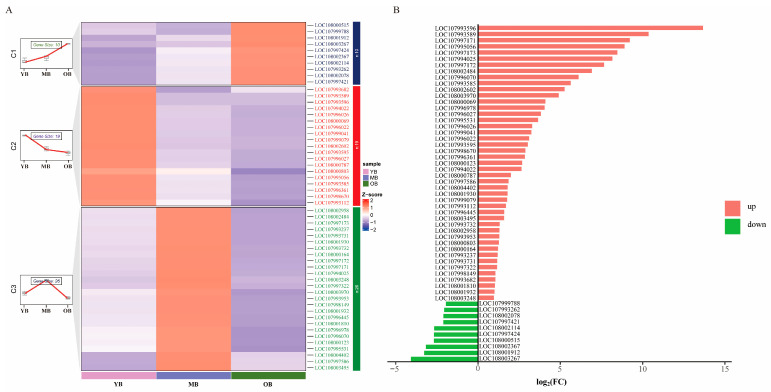
Analysis of DEGs expression patterns comparing different age groups of worker bees. (**A**) Clustered heatmap analysis. Genes with similar expression patterns cluster together in the heatmap, where the color in each square reflects a value normalized across rows of expression data (ranging from −1 to 1). A redder color indicates higher expression levels, while a bluer color indicates lower expression levels. (**B**) FC analysis of DEGs. Red indicates that a gene is upregulated in either the YBs or the MBs but downregulated in the OBs, with green indicating the opposite trend. The *x*-axis, log_2_(FC), represents the FC difference between the YBs and MBs.

After functional annotation filtering, 55 genes were extracted from the 67 DEGs shared among all three contrast groups (MBs vs. YBs, OBs vs. MBs, and OBs vs. YBs) for subsequent analysis (Table S3). Hierarchical clustering (Figure 7A) and fold change (FC) analysis (Figure 7B) revealed that the expression patterns of these genes were significantly correlated with worker bee age, which indicates their potential involvement in aging processes. Based on expression trends (Figure 7A), the 55 genes were clustered into three functionally relevant groups: the first group was aging-upregulated genes, containing 10 genes whose expression levels increased with age and which were lowest in the YBs and highest in the OBs (Figure 7A, blue branch). This group of genes is mainly involved in biological processes such as oxidative stress response, lipid metabolism regulation, and cellular detoxification, such as *glutathione S-transferase 1* (*GST1*, ID 107997421), which is known to directly participate in detoxification processes, neutralizing metabolic wastes and environmental toxins [33]. The second group was aging-downregulated genes, including 19 genes with an opposite expression trend to the first group and namely higher expression in the YBs and gradual downregulation in the MBs and OBs (Figure 7A, red branch). This group of genes is mainly related to the maintenance of cellular functions and structures, such as *Golgi-associated RAB2B interactor protein 3* (*GARIN3*, ID 107993596), which is involved in Golgi-mediated vesicle transport and associated with age-related protein transport regulation in insects [34]. The third group was middle-age-enriched genes, comprising 26 genes that were highly expressed in the MBs and significantly decreased in the OBs (Figure 7A, green branch). This group includes genes related to metabolic activities, such as *major royal jelly protein 3* (*MRJP3*, ID 107997171), which plays an important role in nutrient metabolism and immune regulation, and its peak in the MBs may correspond to the enhanced foraging and defensive activities of middle-aged worker bees [35]. FC analysis further validated these trends: 10 genes were significantly downregulated in the YBs vs. the OBs (log_2_FC: −4.11~−1.99), while 45 genes were upregulated in the YBs vs. the OBs (log_2_FC: 1.01~13.7) (Figure 7B). Combining expression abundance and FC value, 15 core aging-related genes, including *GARIN3*, *APD3* (*apidermin 3*), and *MRJP3*, were screened out (Table 1, Figure S3). These genes exhibited significant expression differences during the aging process of worker bees, which suggests their potential as molecular targets for aging.

### 3.6. Expression Level Analysis of Aging-Related Genes

**Figure 8:**
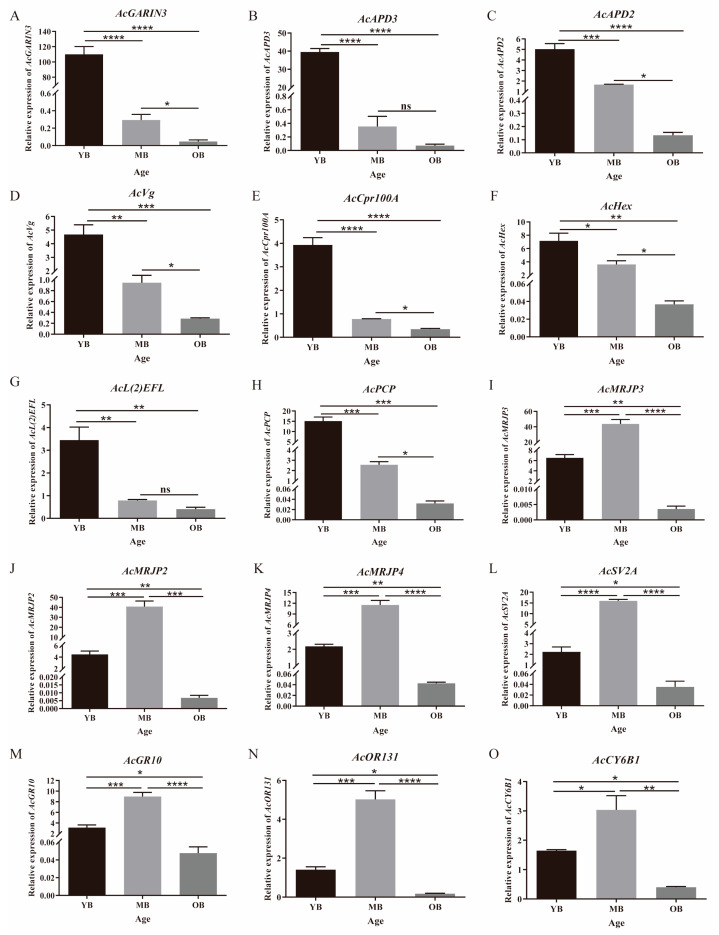
Age-dependent expression patterns of 15 senescence-associated genes identified in *A. cerana* workers were validated by qRT-PCR analysis. Data are presented as mean ± SE (n = 9). * *p* < 0.05, ** *p* < 0.01, *** *p* < 0.001, **** *p* < 0.0001. ns indicates not significant.

The qRT-PCR validation results (Figure 8) demonstrated that the expression trends of the 15 aging-related genes screened from transcriptome sequencing were consistent with the sequencing data across different age groups of worker bees (Table 1, Figure S3). Eight genes (e.g., *AcGARIN3*, *AcAPD3*) were significantly downregulated with the aging process, showing a 3.09–11.2-fold-lower expression in the OBs compared to the YBs (*p* < 0.0001; Figure 8A–H). In contrast, seven genes (e.g., *AcMRJP2*, *AcSV2A*) displayed biphasic expression patterns: their expression peaked in the MBs at 1.88–3.17-fold-higher levels than in the YBs (*p* < 0.01), followed by a significant decline in the OBs to 2.05–10.85-fold lower than the YBs and 2.9–13.5-fold lower than the MBs (*p* < 0.001; Figure 8I–O).

## 4. Discussion

This study revealed significant changes during the aging process of worker bees of *A. cerana* through multidimensional analysis. Morphologically, worker bees exhibit characteristics such as a darkened body color, reduced hairs, and damaged wings as they age, which are consistent with the morphological trends observed in other aging insects, such as the similar changes in body surface bristles in aging *Drosophila melanogaster* [36]. These changes not only affect the appearance of worker bees but may also impair their ability to adapt to the environment. For instance, the reduction in hairs may negatively impact thermoregulation and information perception [37]. In terms of locomotor ability, the number of worker bees in the OB group that reached the feeding platform within 15 s was significantly lower than that of worker bees in the YB and MB groups, which indicates a notable decline in locomotor ability with age. This trend of locomotor decline is similar to the observed decrease in foraging efficiency in aging *A. mellifera* and *Bombus terrestris* [38,39], which suggests that the decline in locomotor function may limit the foraging range and efficiency of worker bees, thereby affecting the productivity of the colony.

Ultrastructural observations have further revealed the structural correlates of worker bee aging. SEM revealed significant changes in the head, thorax, and abdomen structures of worker bees across different ages. During the aging process, worker bees exhibited wear and tear of sensory organs on the head, lodging of thoracic bristles, and varying degrees of cuticle damage. These structural changes directly impair sensory perception, motor efficiency, and cuticular barrier function of worker bees, potentially reducing individual survival rates and task performance. Notably, similar sensory system alterations have been observed in other hymenopterans under urban environmental stress: studies on bees (*Halictus scabiosae*, *Osmia cornuta*) and wasp (*Polistes dominula*) along an urbanization gradient have found that higher temperatures and green space fragmentation lead to changes in the visual and antennal sensory systems. These changes, such as alterations in ommatidia density and diameter, as well as changes in antenna size, may affect sensory perception [40]. This suggests that urban-related stressors may cause sensory degradation similar to that in aging individuals. In a healthy colony, such age-related declines can be mitigated by the continuous emergence of young workers, which maintains a dynamic age structure that supports colony homeostasis. However, senescence may become a critical threat when this balance is disrupted, for instance, due to a skewed age structure (e.g., insufficient emergence of young workers) [41] or accelerated aging and population decline caused by environmental stressors (such as pesticide exposure and nutritional deficiency) [9,42,43,44]. In these cases, the cumulative functional deficits of elderly worker bees may compromise the resilience of the colony, which highlights the context-dependent impact of senescence on honey bee societies.

Additionally, ultrastructural analysis revealed age-related changes in the cuticular structure of worker bees. Compared with YBs, OBs exhibited increased cuticular keratinization and pore density. These changes were accompanied by the downregulation of cuticular protein-encoding genes such as *APD2* and *APD3* (Table 1), which encode structural proteins crucial for maintaining cuticular integrity. APD proteins are key components of insect cuticular cell junctions, promoting intercellular adhesion and forming a physical barrier against environmental stress [45]. Age-dependent downregulation of APD proteins may weaken intercellular adhesion, triggering compensatory keratinization to enhance cuticular rigidity [46,47]. However, excessive keratinization reduces flexibility [48], while increased pore density may reflect disrupted cuticular secretion or impaired lipid barrier synthesis [49]. These alterations collectively compromise the cuticular barrier, exacerbating the susceptibility of old bees to dehydration, pathogen invasion, and chemical stress (e.g., insecticides). Studies on *A. mellifera* have shown that cuticular barrier dysfunction accelerates with age, especially under environmental stress. For example, exposure to sublethal doses of neonicotinoids causes premature downregulation of APD and cuticular thinning [50]. Similarly, dietary protein restriction in bees reduces *APD3* expression and alters keratinization patterns, linking nutrition to cuticular aging [51]. Furthermore, downregulation of *APD2* expression increases the risk of bees being infected by pathogenic organisms [52]. These findings indicate that interactions between molecular regulation (such as *APD* expression) and environmental factors drive age-related physical degradation. Thus, the decline in cuticular barrier function in the OBs may impair their key survival mechanisms. Specifically, cuticular damage increases bees’ sensitivity to water loss, and increased pore density raises the risk of infection. These two factors together may reduce bees’ foraging efficiency and lifespan. Notably, such age-related decline may disrupt colony homeostasis when replacement by new worker bees is insufficient, which is consistent with broader evidence that aging-induced functional deficits become problematic under demographic or environmental stress.

TEM analysis revealed mitochondrial dysfunction as a critical cellular marker of aging. The severity of mitochondrial damage increased with age, while the number of autophagosomes first increased and then decreased. As mitochondria are key sites for energy metabolism, their damage can lead to insufficient energy supply and affect normal cellular functions [53,54]. The dynamic changes in the number of autophagosomes indicate that a significant increase in the autophagosome count in the MBs (20% higher than the YBs) may serve as a compensatory mechanism to delay mitochondrial damage. However, a decrease in autophagic efficiency in the old age group leads to the accumulation of abnormal mitochondria, accelerating the aging process [55]. This finding is consistent with the bidirectional role of autophagy regulation observed in the aging model of *D. melanogaster* [56]. The phased characteristics of autophagosome dynamics in *A. cerana* may provide new insights into the adaptive strategies of their short-lived worker bees. The severe locomotor decline in the OBs directly predicts compromised colony foraging efficiency, while mitochondrial damage and biphasic autophagosome dynamics correlate with metabolic failure, collectively escalating colony vulnerability. Importantly, this biphasic pattern of autophagosome dynamics may serve as a cellular biomarker for colony health deterioration, offering a measurable indicator to predict aging-driven risks in apicultural ecosystems.

Transcriptome analysis revealed that metabolic dysregulation, particularly in redox homeostasis, detoxification, and proteostasis, constitutes a core mechanism driving senescence in worker bees. This is supported by the identification of 2586 DEGs, with 67 shared DEGs potentially playing pivotal roles. These genes are involved in biological processes such as redox reactions and transmembrane transport, as well as signaling pathways related to lifespan regulation, drug metabolism, endoplasmic reticulum protein processing, and lysosomes. For instance, in the lifespan regulation pathway, relevant genes may influence worker bees’ lifespan by regulating their cellular metabolism, antioxidant defense mechanisms, and other mechanisms [57,58]. The cytochrome P450-mediated pathways for drug metabolism and xenobiotic metabolism are crucial for worker bees in coping with external harmful substances. Aging may lead to a decline in the function of these pathways, resulting in the accumulation of harmful substances and accelerated aging [59,60,61]. Abnormalities in endoplasmic reticulum protein processing pathways and lysosome pathways are also observed during worker bee aging, affecting normal protein processing and the clearance of intracellular waste, and thereby interfering with normal cellular physiological activities [62]. Notably, the enrichment of genes in the lysosome pathway is corroborated by the dynamic changes in autophagosome numbers observed through TEM, which suggests that dysfunction of the autophagy–lysosome system may be a core driver of aging in *A. cerana*. Additionally, the downregulation of genes in the glutathione metabolism pathway in the OB worker bees may impair their ability to scavenge reactive oxygen species (ROS) [26,63], which could also be one of the reasons for the exacerbated mitochondrial oxidative damage observed through transmission electron microscopy. Importantly, similar downregulation of mitochondrial and antioxidant gene expression can also be caused by pesticide exposure. A recent study has shown that two common insecticides, imidacloprid and amitraz, can target mitochondrial components such as mitochondrial superoxide dismutase (MnSOD) and inhibit the expression of cytosolic antioxidant genes (such as DUOX and CuZnSOD), leading to the accumulation of hydrogen peroxide (H_2_O_2_) and impairment of antioxidant defense mechanisms [64]. These changes mirror the age-related mitochondrial damage and redox imbalance identified in our study. This comparison supports the hypothesis that environmental stressors, such as pesticide exposure, may exacerbate age-related declines in mitochondrial function and antioxidant defenses, thereby accelerating senescence.

Fifteen core aging-related targets were identified through differential expression gene screening and validation (Table 1, Figure 8), covering nutritional metabolism, cellular regulation, and sensory functions. Among them, the major royal jelly protein (*MRJP*) genes, including *MRJP2*, *MRJP3*, and *MRJP4*, were significantly downregulated in aged worker bees, potentially affecting their immune and metabolic homeostasis by reducing the supply of nutritional factors. This is similar to the mechanism by which royal jelly proteins regulate the lifespan in *A. mellifera* [30,35]. However, we recognize that *MRJP* downregulation could also occur under conditions of nutritional deprivation or immune stress, which indicates a need to contextualize these markers with additional physiological data. In addition to *MRJPs*, two genes related to nutritional storage in *A. cerana*, *Vg* and *Hex*, were also downregulated with age, with Vg depletion potentially weakening antioxidant defenses and immune homeostasis [65,66], while the low expression of Hex may be related to their decreased energy demands [67]. While Vg is widely recognized as an aging marker, its expression can also be modulated by juvenile hormone levels and reproductive status [19,20], which suggests complex interactions between aging and other physiological processes. Furthermore, cellular homeostasis markers *GARIN3* and *L2EFL* exhibited suppressed expression in the OBs, potentially accelerating aging via disrupted Golgi function or cell cycle regulation [34,68]. The expression dynamics of *CYP6B1* suggest an age-related decline in its ability to metabolize environmental toxins, which potentially exacerbates the accumulation of oxidative damage [69]. This gene’s dual involvement in aging and detoxification processes complicates the distinction between age-related decline and environmental stress effects. To address this, we recommend longitudinal monitoring of *CYP6B1* expression alongside pesticide residue analysis in hive matrices, which would enable correlation of molecular changes with specific environmental exposures. The gustatory receptor gene *GR10* and odorant receptor gene *OR131* play roles in the recognition and selection of food sources by worker bees, and changes in their expression levels may affect their foraging efficiency and survival ability [70]. While foraging-related gene expression changes are clearly age-associated in *A. cerana*, variations in floral resource availability and pesticide contamination could confound these patterns [71,72]. Taken together, these candidate markers collectively provide a molecular framework for monitoring aging progression in *A. cerana*, but their utility depends on integration with behavioral, physiological, and environmental data. This multi-dimensional approach will help distinguish aging-specific changes from general stress responses, enhancing the reliability of these markers for predicting colony health risks and guiding senescence intervention strategies.

Although this study comprehensively analyzed the characteristics of worker bee senescence from multiple dimensions, and these characteristics showed a strong age-dependent progression, it still has limitations such as a limited sample size that may constrain the generalizability of the proposed senescence-related traits and an experimental design focused solely on age-related changes without addressing environmental interactions (e.g., pesticide exposure, pathogen load), which is critical for field applications. Future research should prioritize validating these candidate senescence markers (e.g., WWI, *APD3* expression) under ecologically realistic conditions to assess their sensitivity to environmental stressors, investigate synergistic effects of extrinsic factors on aging trajectories, and apply gene-editing tools to confirm functional links between marker dynamics and colony health outcomes. These advancements will refine the markers into a predictive framework for early detection of aging-driven colony risks, ultimately supporting targeted interventions and providing a more solid scientific basis for bee health management and the field of senescence biology.

## 5. Conclusions

*A. cerana* worker bees exhibit multi-scale age-related degradation. Morphologically, worker bees undergo darkening of their body color, loss of hairs, wing damage, and decreased locomotor capacity. These morphological changes directly reflect the decline in physiological functions and impair the colony’s foraging efficiency and food reserve capacity. Structurally, epidermal damage and pore formation are associated with the downregulation of *APD2* and *APD3* genes, while mitochondrial damage and dynamic changes in autophagy efficiency suggest an imbalance in energy metabolism and cellular clearance mechanisms. At the molecular level, the differential gene regulatory network is significantly enriched in pathways related to lifespan regulation, glutathione metabolism, drug metabolism, endoplasmic reticulum protein processing, and lysosomal pathways. Among them, key targets such as *MRJP2*, *GARIN3*, and *CYP6B1*, which regulate nutrient metabolism, organelle function, and toxin metabolism, hold potential as targets for senescence intervention. Although this study systematically reveals the cross-scale senescence characteristics of worker bees and preliminarily identifies these characteristics as potential senescence markers, the interactions between these senescence characteristics and environmental stressors need to be verified through field control experiments and gene-editing methods. Collectively, this study provides a theoretical basis for optimizing honey bee health management strategies and exploring the senescence mechanisms of social insects.

## Figures and Tables

**Table 1:** Screening of aging-related candidate targets and their functional descriptions.

Gene_ID	Gene Annotation	Average Expression Level			YB/OB	Difference Multiplier	Order	Gene_Description
		YB	MB	OB		log_2_(FC)		
107993596	GARIN3	3347.207	4.524578	0.249664	13,406.84	13.71	1	Golgi-associated RAB2B Interactor protein 3
107993589	APD3	484.3711	3.640512	0.359029	1349.115	10.43	2	Apidermin 3
107997171	MRJP3	21,312.96	109,807.9	34.8865	610.923	9.25	3	Major royal jelly protein 3
107995056	Hex	1137.21	375.6247	2.319312	490.322	8.94	4	Hexamerin
107997173	MRJP2	5295.705	18,781.23	14.57883	363.2462	8.50	5	Major royal jelly protein 2
107997172	MRJP4	21,093.77	99,826.85	102.1779	206.4416	7.69	6	Major royal jelly protein 4
108002484	SV2A	92.92831	333.0887	0.754609	123.1476	6.94	7	Synaptic vesicle glycoprotein 2A
107996070	GR10	25.42094	60.81404	0.360529	70.51007	6.14	8	Gustatory receptor 10
107993585	APD2	1964.391	632.1537	38.80771	50.61858	5.66	9	Apidermin 2/cuticle protein 1
108002602	PCP	113.7687	11.70071	0.297192	39.37086	5.27	10	Pupal cuticle protein
108003970	CYP6B1	99.4837	258.644	8.422693	30.70799	4.94	11	Cytochrome P450 6B1
107999079	Cpr100A	29.13097	4.140551	1.183832	24.60733	4.62	12	Cuticular protein 100A
108000069	Vg	2362.827	437.8775	134.7151	17.53944	4.13	13	Vitellogenin
107996978	OR131	18.87142	43.73428	1.119135	16.86251	4.08	14	Odorant receptor 131
107996027	L(2)EFL	2249.161	516.4503	155.335	14.47942	3.86	15	Protein lethal (2) essential for life

Note: YB, young bee; MB, middle-aged bee; OB, old bee. YB/OB, gene expression levels in YB/OB.

## Data Availability

The raw transcriptomic sequencing data for the head tissues of *A. cerana* worker bees across different age groups have been deposited in the NCBI Sequence Read Archive (SRA) under accession number PRJNA1234368.

## Supplementary Materials

The following supporting information can be downloaded at: https://www.mdpi.com/article/10.3390/insects16090902/s1. Figure S1. Schematic diagram of the experimental workflow for multi-dimensional analysis of aging characteristics in *Apis cerana* worker bees. In the figure, T and H represent temperature and humidity, respectively; dpe stands for days post-eclosion. Each experimental project was conducted with at least three biological replicates, and each replicate was from a different colony. Figure S2. Stereomicroscope observation of morphological characteristics in head tissues of worker bees at different age groups. (A) Schematic diagram for measuring the sagittal plane (red dashed line) and coronal plane (blue dashed line) dimensions of worker bee brains; (B) Sagittal plane length and coronal plane width among groups. Data were presented as mean ± SEM (n = 20). The results of one-way ANOVA revealed no significant difference among groups (*p* > 0.05). Figure S3. Clustered heat man of age-dependent expression of 15 senescence-associated genes in *A. cerana* workers. Table S1. Survival dynamics of marked *Apis cerana* worker bees in natural colony conditions. Table S2. Information of specific amplification primers for real-time quantitative PCR. Table S3. List of DEGs information shared by worker bees across different age groups.

## Abbreviations

The following abbreviations are used in this manuscript:

YB	Young bee
MB	Mid-aged bee
OB	Old bee
SEM	Scanning electron microscopy
TEM	Transmission electron microscopy
DEGs	Differentially expressed genes
SE	Mean ± standard error of the mean
FPKM	Fragments per Kilobase of Exon per million Mapped Fragments
Log_2_FC	log_2_Fold Change
GO	Gene Ontology
KEGG	Kyoto Encyclopedia of Genes and Genomes
