# Supplementary material for: Multidimensional Profiling of Senescence in Eastern Honey Bee, Apis cerana (Hymenoptera: Apidae), Workers: Morphology, Microstructure, and Transcriptomics
Source: Insects. 2025 Aug 28;16(9):902. doi: 10.3390/insects16090902 (PMC12470740; doi:10.3390/insects16090902)
Supplement: Supplementary file 1 [file insects-16-00902-s001.zip › Supplementary Figure S1.pdf]

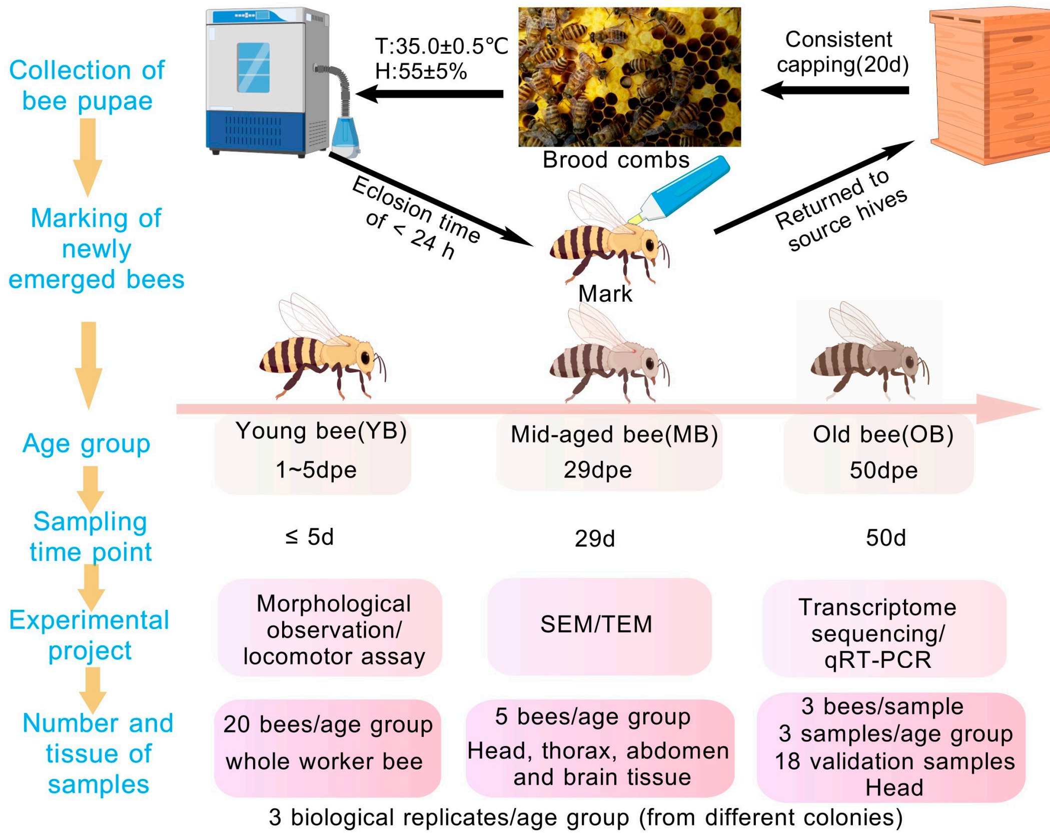


**Figure S1. Schematic diagram of the experimental workflow for multi-dimensional analysis of aging characteristics in *Apis cerana* worker bees.** In the figure, T and H represent temperature and humidity, respectively; dpe stands for days post-eclosion. Each experimental project was conducted with at least three biological replicates, and each replicate was from a different colony.
