# Supplementary material for: Multidimensional Profiling of Senescence in Eastern Honey Bee, Apis cerana (Hymenoptera: Apidae), Workers: Morphology, Microstructure, and Transcriptomics
Source: Insects. 2025 Aug 28;16(9):902. doi: 10.3390/insects16090902 (PMC12470740; doi:10.3390/insects16090902)
Supplement: Supplementary file 1 [file insects-16-00902-s001.zip › Supplementary Figure S2.pdf]

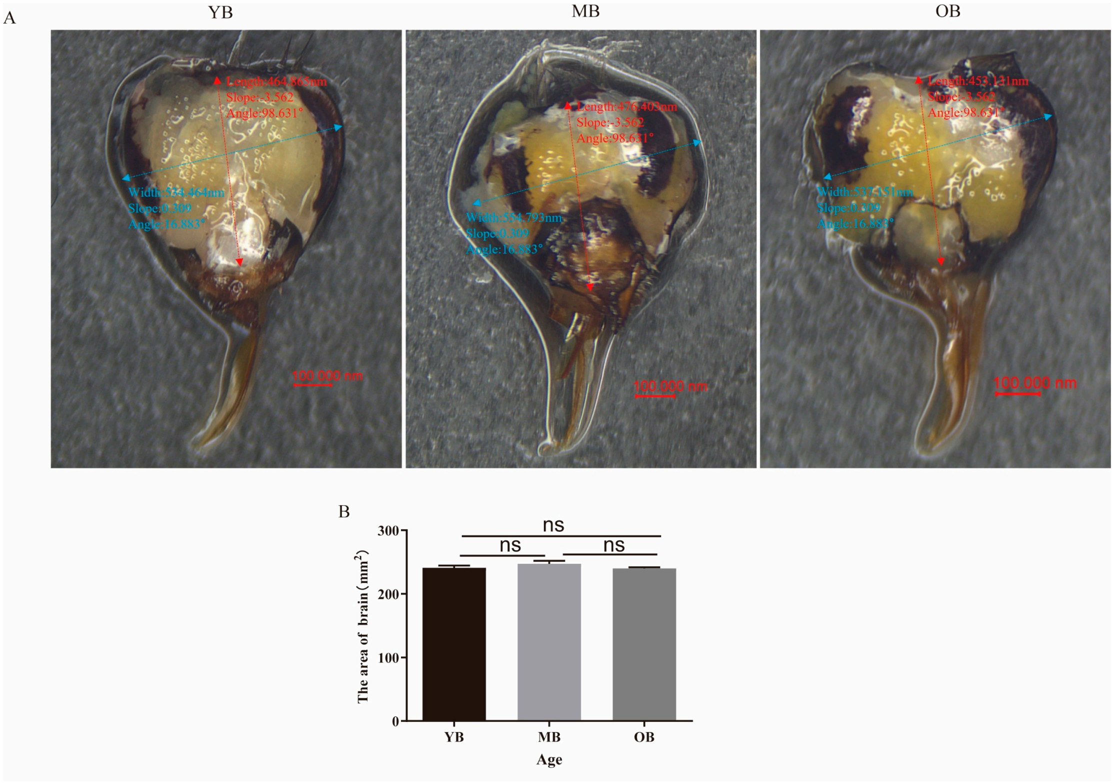

Figure S2. Stereomicroscope observation of morphological characteristics in head tissues of worker bees at different age groups. (A) Schematic diagram for measuring the sagittal plane (red dashed line) and coronal plane (blue dashed line) dimensions of worker bee brains; (B) Sagittal plane length and coronal plane width among groups. Data were presented as mean ± SEM (n=20). The results of one-way ANOVA revealed no significant difference among groups ($p > 0.05$).
