# Supplementary material for: Multidimensional Profiling of Senescence in Eastern Honey Bee, Apis cerana (Hymenoptera: Apidae), Workers: Morphology, Microstructure, and Transcriptomics
Source: Insects. 2025 Aug 28;16(9):902. doi: 10.3390/insects16090902 (PMC12470740; doi:10.3390/insects16090902)
Supplement: Supplementary file 1 [file insects-16-00902-s001.zip › Supplementary Figure S3.pdf]

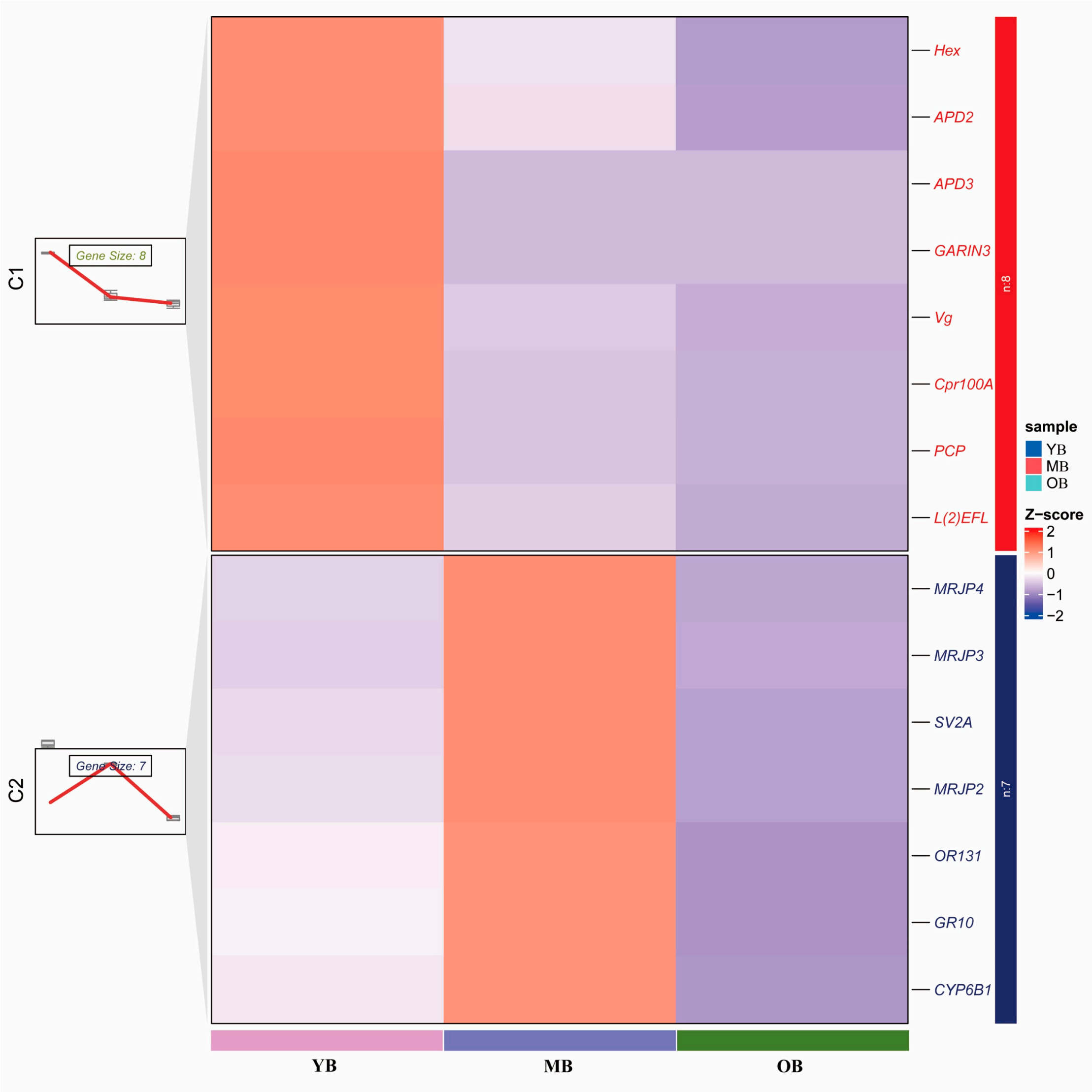

Figure S3. Clustered heat man of age-dependent expression of 15 senescence-associated genes in *A. cerana* workers.
