# Supplementary material for: Multidimensional Profiling of Senescence in Eastern Honey Bee, Apis cerana (Hymenoptera: Apidae), Workers: Morphology, Microstructure, and Transcriptomics
Source: Insects. 2025 Aug 28;16(9):902. doi: 10.3390/insects16090902 (PMC12470740; doi:10.3390/insects16090902)
Supplement: Supplementary file 1 [file insects-16-00902-s001.zip › Supplementary Table S1.pdf]

**Table S1** Survival dynamics of marked *Apis cerana* worker bees in natural colony conditions

| **Days post-emergence (dpe)** | **Age group** | **Colony 1** Bees survived (% survival) | **Colony 2** Bees survived (% survival) | **Colony 3** Bees survived (% survival) | **Mean survived bees** (mean ± SE) | **Mean survival rate** (%, mean ± SE) |
|---|---|---|---|---|---|---|
| 5 dpe | Young bees (YB) | 390 (97.5%) | 393 (98.3%) | 384 (96.0%) | 389.00 ± 2.65 | 97.27 ± 0.67 |
| 29 dpe | Mid-aged bees (MB) | 295 (73.8%) | 266 (66.5%) | 312 (78.0%) | 291.00 ± 13.43 | 72.77 ± 3.36 |
| 50 dpe | Old bees (OB) | 98 (24.5%) | 77 (19.3%) | 91 (22.8%) | 88.67 ± 6.17 | 22.17 ± 1.54 |
| 60 dpe | Senescent bees (SB) | 18 (4.5%) | 11 (2.8%) | 13 (3.3%) | 14.00 ± 2.08 | 3.53 ± 0.52 |

Note: Initial marked bees: 400 per colony × 3 colonies = 1,200 bees; dpe stands for days post-eclosion.
