# Supplementary material for: Multidimensional Profiling of Senescence in Eastern Honey Bee, Apis cerana (Hymenoptera: Apidae), Workers: Morphology, Microstructure, and Transcriptomics
Source: Insects. 2025 Aug 28;16(9):902. doi: 10.3390/insects16090902 (PMC12470740; doi:10.3390/insects16090902)
Supplement: Supplementary file 1 [file insects-16-00902-s001.zip › Supplementary Table S2.pdf]

Table S2 Information of specific amplification primers for real-time quantitative PCR

Gene	Primer sequence	Product length (bp)
AcGARIN3	F: ATCGTCCTTGTCGCTGCCTT	97
	R: CACCTAACACCACTGCTCCAT	
AcAPD3	F: TGCTTGACCATCCTCGTAGC	164
	R: AGGTGTCCAGCAAGAAGAGC	
AcMRJP3	F: GTTGGCTGCGTGAATGAACA	126
	R: TCTGCCGGATTGTGGAAGAT	
AcHex	F: CAGTCCCGGGAAGTGGTAAC	265
	R: GTGGGTTGACCTTGGTGGAA	
AcMRJP4	F: AGTGGTTGTTGTTGATGGCA	276
	R: AAGAGGAAGGCACACCATCG	
AcSV2A	F: CGACTACCGGTGTTGTTGGA	200
	R: GTTTACTACGCAAGCACCGC	
AcGR10	F: CATGACAGCTCATCAGGCGA	161
	R: CGACGCGTATTAAACCAGCA	
AcAPD2	F: TTTGAAAGGGAACGCCGAGG	151
	R: GGGCGGCTACAACTGCT	
AcCYP6B1	F: ACCCACTTACGCCATTCACA	130
	R: TGGTCCGTGTCCAAAAGGTA	
AcVg	F: GCACATTGACCAGTTTGGGC	144
	R: GCATGGACTGCTGGATACGA	
AcCpr100A	F: CAATCACCCCAACAAGCTGC	87
	R: ATCCTCTTGCGAATAGGCGG	
AcL(2)EFL	F: GACCTGGATCGTCCACATCG	163
	R: GCAGAAGTTCACCCCAAGGT	
AcMRJP2	F: AGCTGTTGGTTGTTGGAACG	265
	R: TAACAAGTCGGTCACACCTCC	
AcPCP	F: AGTGGTATCCTGGGGCGTAT	199
	R: GCATAGTTTCCAGGGGCGTA	
AcOR131	F: TGGCTATAAGTCTGCATCGAGT	250
	R: ACACATGCCTTTGAGCTTCG	
AcActin	F: TCCTGCTATGTATGTCGC	301
	R: GGTTGCCATTTCCTGTTC	

Note: *Actin* is reference gene.
