# Supplementary material for: Multidimensional Profiling of Senescence in Eastern Honey Bee, Apis cerana (Hymenoptera: Apidae), Workers: Morphology, Microstructure, and Transcriptomics
Source: Insects. 2025 Aug 28;16(9):902. doi: 10.3390/insects16090902 (PMC12470740; doi:10.3390/insects16090902)
Supplement: Supplementary file 1 [file insects-16-00902-s001.zip › Supplementary Table S3.pdf]

Table S3 List of DEGs information shared by worker bees across different age groups

Gene_ID	Gene Annotation	Average expression level YB	MB	OB	YB/OB	log_2(FC)	gene_description
107999788	TAT	5.488787	2.324668	21.82393	0.251503111	-1.99138	tyrosine aminotransferase
108000515	ELOVL	2.971339	0.856671	19.60261	0.151578742	-2.722466	elongation of very long chain fatty acids protein
108003267	FAR	3.326999	8.664782	57.60944	0.057750928	-4.24792813	putative fatty acyl-CoA reductase
108002078	GPCRs	5.461385	11.55419	24.19816	0.225694191	-2.150028	G-protein coupled receptor Mth2
107993262	AAH	11.14896	22.92637	47.56809	0.234378942	-2.092044	fatty-acid amide hydrolase 2-B
108001912	LAC5	141.6499	443.7369	1404.64	0.10084424	-3.310685	laccase-5
107997421	GST1	28.31246	60.38609	125.6088	0.22540195	-2.150175	glutathione S-transferase 1
107997424	GST1L	0.841365	2.689758	5.546789	0.151685054	-2.722232	glutathione S-transferase 1-1-like
108002114	KCP	298.1208	881.1706	1940.82	0.15360556	-2.702486	kielin/chordin-like protein
108002367	PLRP2	18.50971	70.85629	170.4529	0.108591355	-3.203606	pancreatic lipase-related protein 2
107993596	GARIN3	3347.207	4.524578	0.249664	13406.83723	13.71068131	Golgi-associated RAB2B interactor protein 3
107993589	APD3P	484.3711	3.640512	0.359029	1349.115369	10.42547	apidermin 3
107996361	CI	1344.797	552.8846	183.2757	7.337562267	2.867594165	chymotrypsin inhibitor
107996027	L(2)EFL	2249.161	516.4503	155.335	14.47942168	3.85593	protein lethal(2)essential for life
108000069	Vg	2362.827	437.8775	134.7151	17.53943533	4.13253	vitellogenin
107995056	Hex	1137.21	375.6247	2.319312	490.3220437	8.93759	hexamerin
107993585	APD2	1964.391	632.1537	38.80771	50.61857793	5.6616	apidermin 2/cuticle protein 1
107996026	L(2)EF	2507.495	600.944	249.8816	10.0347297	3.321928095	protein lethal(2)essential for life
107996022	L(2)EF	839.997	196.8138	95.51352	8.79453535	3.044522438	protein lethal(2)essential for life

108000803	Sirt1	336.9408	242.4349	101.1714	3.33039675	1.700439718	NAD-dependent protein deacetylase Sirt1
107993112	Sirt2	944.2733	622.1164	381.9981	2.471931906	1.301029996	NAD-dependent protein deacetylase Sirt2
107998670	VAT1L	137.8681	53.64339	18.31463	7.527759883	2.877594165	synaptic vesicle membrane protein VAT-1 homolog-like
107994022	CRYAA	502.4453	87.46532	13.65652	6.40465805	2.678429	alpha-crystallin A chain
108002602	PCP	113.7687	11.70071	0.297192	39.37086	8.580085	pupal cuticle protein-like
107999041	sNPF	53.24809	11.61819	5.476265	9.723431653	3.2818	short neuropeptide F-like
107999079	Cpr100A	29.13097	4.140551	1.183833	24.60733848	4.621766	Cuticular protein 100A:Insect cuticle protein
108000787	PBP	27.09234	6.554948	1.596739	4.105208137	2.038112	Pheromone-binding protein-related protein
107993595	EC4A	388.9587	108.3032	46.69869	8.329112763	3.058006	endochitinase A1-like
107993682	CP7	4.913743	1.092153	2.31247	2.124889746	1.08787	cuticle protein 7-like
107997171	MRJP3	21312.96	109807.9	34.8865	610.9229937	9.253336	major royal jelly protein 3-like
107997173	MRJP2	5295.705	18781.23	14.57883	363.2461862	8.504423	major royal jelly protein 2-like
107994025	MRJP7	6280.037	37814.87	21.36313	293.9661816	8.199509	major royal jelly protein 7-like
107997172	MRJP4	21093.77	99826.85	102.1779	206.4415839	7.689433	major royal jelly protein 4-like
107996978	OR131	18.87142	43.73428	1.119135	16.86250644	4.075187	odorant receptor 131
107996070	GR10	25.42094	60.81404	0.360529	70.51006965	6.14007	gustatory receptor 10
108003495	H1	11.22444	75.52339	24.62582	3.06683813	1.616375	histone H1
107997586	NPC2	2.537354	26.63807	7.200045	3.699708461	1.887525	NPC intracellular cholesterol transporter 2
108003970	CYP6B1	99.4837	258.644	8.422693	11.81138774	3.562423	cytochrome P450

							6B1-like
108000123	PFC0760c	42.54249	96.82514	6.522108	6.522812593	2.705525	protein PFC0760c
107995531	SPE5L	29.64774	64.91097	2.313715	12.81390934	3.678072	protein spaetzle 5-like
108002484	SV2A	92.92831	333.0887	0.754609	123.1476246	6.944029	synaptic vesicle glycoprotein2A
108003248	GBA	283.7964	1998.412	141.9322	1.99952122	0.999521	lysosomal acid glucosylceramidase
107997322	ABCC2	6.256425	31.84764	2.748725	2.276119139	1.187625	ATP-binding cassette sub-family C member Sur
107996445	Tret1	23.17029	54.13508	7.532079	3.076214976	1.621325	facilitated trehalose transporter Tret1-like
107993732	Map21	78.56371	234.927	30.88986	2.543349301	1.346054	protein mab-21
107993237	FMO	11.17276	28.85602	4.793689	2.330722701	1.220448	flavin-containing monooxygenase FMO GS-OX-like 4
108000164	SDH	35.461	98.82657	14.94595	2.372616178	1.246804	succinate dehydrogenase
107993731	Map21L	9.873535	25.21349	4.291089	2.300939231	1.202429	protein mab-21-like
107998149	BOK	18.84327	39.83178	8.824414	2.135356257	1.094179	bcl-2-related ovarian killer protein
108001930	MSF3	7.835438	24.02991	2.211638	3.542820663	1.824509	MFS3 major facilitator superfamily transporter 3
108001810	POD	49.89919	100.4191	23.86704	2.090715831	1.064463	peroxidasin
108002958	DJC22	90.09177	220.2221	35.63371	2.528273709	1.337799	dnaJ homolog subfamily C member 22
107993953	TIAL1	1.510566	3.3916	0.603743	2.50199969	1.322183	nucleolysin TIAR
108001932	ABCE1	3.887555	8.205846	1.893683	2.052907602	1.036906	ATP-binding cassette sub-family E member 1
108004402	CYP6A	2.81642	24.37323	6.830738	3.568168959	1.834882	probable cytochrome P450 6a
